# Deep, multi-stage transcriptome of the schistosomiasis vector *Biomphalaria glabrata* provides platform for understanding molluscan disease-related pathways

**DOI:** 10.1186/s12879-016-1944-x

**Published:** 2016-10-28

**Authors:** Nathan J Kenny, Marta Truchado-García, Cristina Grande

**Affiliations:** 1 Department of Zoology, University of Oxford, Oxford, OX1 3PS UK; 2 Present Address: Simon FS Li Marine Science Laboratory, School of Life Sciences and State Key Laboratory of Agrobiotechnology and Soyabean Research Centre, The Chinese University of Hong Kong, Shatin, Hong Kong; 3 Departamento de Biologia Molecular and Centro de Biologia Molecular “Severo Ochoa” (CSIC, Universidad Autonoma de Madrid), Madrid, Spain; 4 Present Address: Departamento de Biologia, Universidad Autonoma de Madrid, Campus de Cantoblanco, 28049 Madrid, Spain

**Keywords:** *Biomphalaria glabrata*, Transcriptome, Disease response, Schistosomiasis, Bilharzia, Gastropoda, Planorbidae

## Abstract

**Background:**

The gastropod mollusc *Biomphalaria glabrata* is well known as a vector for the tropical disease schistosomiasis, which affects nearly 200 million people worldwide. Despite intensive study, our understanding of the genetic basis of *B. glabrata* development, growth and disease resistance is constrained by limited genetic resources, constraints for which next-generation sequencing methods provide a ready solution.

**Methods:**

Illumina sequencing and *de novo* assembly using the Trinity program was used to generate a high-quality transcriptomic dataset spanning the entirety of *in ovo* development in schistosomiasis-free *B. glabrata*. This was subjected to automated (KEGG, BLAST2GO) and manual annotation efforts, allowing insight into the gene complements of this species in a number of contexts.

**Results:**

Excellent dataset recovery was observed, with 133,084 contigs produced of mean size 2219.48 bp. 80,952 (60.8 %) returned a BLASTx hit with an *E* value of less than 10^-3^, and 74,492 (55.97 %) were either mapped or assigned a GO identity using the BLAST2GO program. The CEGMA set of core eukaryotic genes was found to be 99.6 % present, indicating exceptional transcriptome completeness. We were able to identify a wealth of disease-pathway related genes within our dataset, including the Wnt, apoptosis and Notch pathways. This provides an invaluable reference point for further work into molluscan development and evolution, for studying the impact of schistosomiasis in this species, and perhaps providing targets for the treatment of this widespread disease.

**Conclusions:**

Here we present a deep transcriptome of an embryonic sample of schistosomiasis-free *B. glabrata,* presenting a comprehensive dataset for comparison to disease-affected specimens and from which conclusions can be drawn about the genetics of this widespread medical model. Furthermore, the dataset provided by this sequencing provides a useful reference point for comparison to other mollusc species, which can be used to better understand the evolution of this commercially, ecologically and medically important phylum.

**Electronic supplementary material:**

The online version of this article (doi:10.1186/s12879-016-1944-x) contains supplementary material, which is available to authorized users.

## Background

**Fig. 1:**
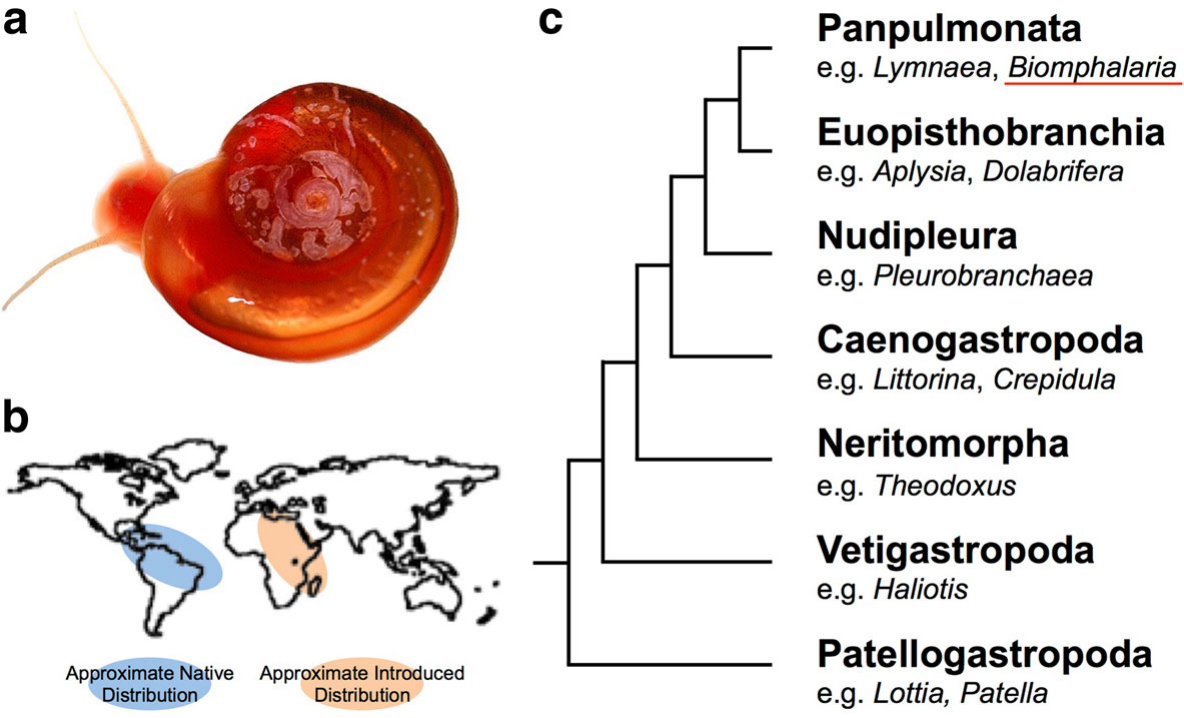
**a** Adult albino *Biomphalaria glabrata* (image courtesy of Lewis et al., [32] CC BY 2.5). **b** Approximate native and introduced distribution of *B. glabrata* worldwide [44]. **c** Gastropod phylogeny (after [30]) showing position of *B. glabrata* (underlined in red) within the Panpulmonata

*Biomphalaria glabrata* ([47], image Fig. 1a) is a neotropical species, native to the Caribbean and northern parts of South America, but now widespread throughout tropical areas of North, Central and South America [44]. It belongs to a species complex found in both the Old and New Worlds, and *B. glabrata* itself has now spread to Africa and the Middle East (Fig. 1b). The genus *Biomphalaria* (comprising approximately 34 species) and *B. glabrata* itself are perhaps best known for their role in the transmission of the parasites which cause schistosomiasis (bilharzia), a disease found in 70 countries and infecting approximately 200 million people worldwide, with a further seven hundred million people at risk [15, 17]. While it is not the only snail vector of this disease, *B. glabrata* is the best studied, with a long history of investigation, dating back over 50 years [41].

The genetic sequences of parasites which can cause schistosomiasis - *Schistosoma mansoni* [7], *Schistosoma japonicum* [53] and *Schistosoma haematobium* [55] have been available for several years, with *B. glabrata* the intermediate host of *S. mansoni.* The sequencing of the genome of *B. glabrata* itself is still in progress, despite being identified as a priority target for genomic sequencing as early as 2004 [45], although preliminary data is now released on GenBank (PRJNA12879/PRJNA290623). Despite the ever-growing availability of next-generation sequencing, gastropod molluscs, which represent a sizable proportion of all animal diversity with over 40,000 extant species, are still under-represented by published, publically available genome sequences. This has hamstrung our attempts to understand the genetic and molecular parasite/host interactions that occur in the course of schistosomiasis.

Much work has already been conducted into the genetic and molecular responses made by *B. glabrata* to infection, in the hope of identifying potential targets for treating and mitigating the effects of schistosomiasis. (For examples, see [3, 8, 9, 12, 18, 25–27, 33, 34, 38–40, 43, 46, 52, 54, 58]). This work has revealed several molecular families involved in immune response within *B. glabrata* and the Mollusca as a whole, but has been hamstrung by the limitations of EST- and specific target gene-based approaches. Presently existing public sequence resources for *B. glabrata* remain limited, despite a range of prior efforts, including EST based [35, 36] BAC [2] and transcriptomic datasets [13, 14] with the latter, most recent resource identifying 30,206 transcripts with at least one associated gene ontology (GO) term from adult samples. While a raft of prior sequencing work has been undertaken in this organism (summarized in [13]) it has generally lacked the scope which modern next-generation sequencing methods can bring to bear, and as such no suitable reference genomic or transcriptomic resource covering the course of the *in ovo* development of *B. glabrata* exists.

A high-quality, well-assembled and annotated transcriptome, in the absence of a complete genomic resource, is therefore vitally important to provide a framework for future investigation. Next-generation sequencing and *de novo* assembly algorithms have progressed to the point where the construction of highly informative datasets is straightforward, cost-effective and of much utility to laboratories worldwide [37, 48], and such an approach has immediate utility for the *B. glabrata* community in a range of contexts. Furthermore, in the absence of a *B. glabrata* genome, which has been noted as problematic to assemble in recent literature [13] it will allow for the first time comprehensive investigation of the effects of parasite infection in comparative studies involving *B. glabrata*, *Homo sapiens* and schistosome species, acting as a firm reference point for such work.

The Mollusca also remain relatively undersampled, with the sequences of two bivalves, the oysters *Pinctada fucata* and *Crassostrea gigas* [51, 57], a single cephalopod [4] and two gastropods (Patellogastopoda), *Lottia gigantea* and *Patella vulgata* [29, 49] publically available. Seven other species of mollusc have genomic sequence data available from the NCBI genome site, although these have yet to be formally published. Species with published genomes are also relatively far removed from the Panpulmonata, which, as can be seen in Fig. 1c, is separated phylogenetically from their closest sequenced relatives in the Patellogastopoda by several major branching events.

Molluscan transcriptomic resources are more widely available, and, for example, another pulmonate species, *Radix balthica,* has been the target of a *de novo* transcriptomic approach [16], and has yielded reasonable results, albeit with a relatively low mean contig length (536 bp). A number of EST and transcriptomic analyses have been performed in this phylum and in this species (e.g. [14]), but in general our knowledge of the transcriptional repertoire of the wider Mollusca remains depauperate.

The dataset presented in this paper will therefore stand as an excellent resource for the investigation of a range of traits within the Mollusca, and more pertinently for human health, for efforts investigating the progression of schistosomiasis within *B. glabrata*. As demonstrated in this paper, the pathways involved in growth and disease response are well represented and in many cases likely complete in this dataset, allowing the firm inference of the true responses of *B. glabrata* to infection, and providing valuable insight into these networks within the Mollusca as a whole.

## Methods

### Animal culture and RNA extraction

**Fig. 2:**
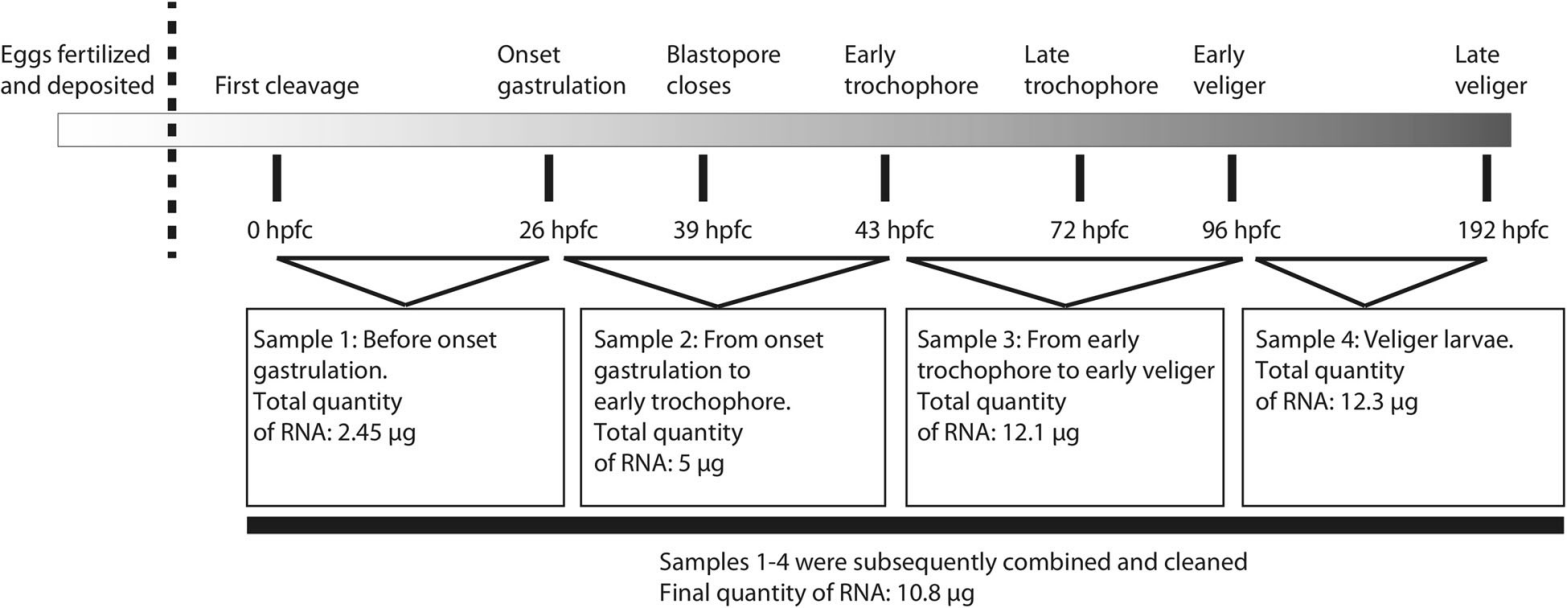
Summary of staged RNA sources and quantities, and their relationship to known events in *B. glabrata* embryogenesis [10]. hpfc: hours post first cleavage

*B. glabrata* M-line strain were sourced from BRI Resources, NIAID, NIH and cultured in the laboratory as described in Grande and Patel [24]. M-line strains were used due to their history of use in laboratory-based and sequencing studies, and known susceptibility to schistosomiasis. The RNA used to prepare this library was obtained from early cleavage stage embryos up through to advanced veliger larval stages until the point prior to hatching (Fig. 2). Total RNA was immediately extracted after collection in both embryonic and larval samples, which were mixed as shown in Fig. 2, and these samples were prepared using Trizol following the manufacturer’s instructions (Life Technologies), with tissue homogenized using sterile plastic pestles. RNA concentration and quality were quantified with a Nanodrop spectrophotometer. Samples were mixed according to the proportions seen in Fig. 2, with pooled sequencing chosen due to fiscal constraints, and to ensure maximum sequencing depth from the single lane available. The sample for sequencing was stored at -80 °C and shipped on dry ice to the sequencing provider.

### RNA sequencing, quality control and assembly

A single lane was sequenced on the Illumina HiSeq platform (HiSeq2000) at the Sequencing Service of the Biomedicine and Biotechnology Institute of Cantabria (IBBTEC; Santander, Spain) with 101 bp, paired end, read length. The resulting reads were made available to us in fastq format on an external server, and were downloaded for local analysis, with statistics as detailed in Table 1. Initial assessments of the quality of read data were performed using FastQC [6]. Reads were assembled using Trinity r2012-06-08 [23] with all default settings. After assembly, contigs less than 500 bp in length were removed from our dataset, and final assembly metrics were determined using a perl script available from the authors upon request. CEGMA [42] was run on the TRUFA platform [31].

**Table 1:** Basic read data: Summary statistics pertaining to reads used in the present study

Platform	Illumina HiSeq
Number of Read Pairs	52,648,074
Read Length (bp)	101
Insert Size (bp, nominal)	300
GC content (%)	39

### Functional annotation and KEGG pathway assignment

We searched for homologues and annotated our dataset according to GO terms with BLAST2GO 2.5.0 (web start) queries against the nr database [11, 21]. *D. melanogaster* GO term distribution in that species’ genome was downloaded from B2GO-FAR [22] and analysed with the Combined Graph function embedded in BLAST2GO. For assignment to KEGG pathways, the KAAS-KEGG Automatic Annotation Server (http://www.genome.jp/tools/kaas/) was used to process data using the single-directional best-hit (SBH) option, the default (60) BLAST bit score threshold and the hsa, mmu, xla, dre, cin, spu, dme, ame, cel, smm, nve and tad datasets. Where genes were not found in our dataset, BLASTp of known orthologues of genes was performed against all spiralian (= lophotrochozoan *sensu* [20]) sequences in the nr database. Where no possible spiralian orthologue was found, genes were noted as ‘absent in Molluscs’, otherwise these were coded as ‘Absent’ in our dataset.

### Manual annotation

tBLASTn [5] using gene sequences of known homology (sourced from the NCBI nr database) as queries against standalone databases created on a local server using BLAST 2.2.29+ manually identified genes of particular interest to this study. These putatively identified sequences were then reciprocally BLASTed against the total NCBI nr database using BLASTx for confirmation of identity. Where identity was uncertain after reciprocal BLAST, diagnostic residues or domains were used to infer identity as noted in text.

## Results and discussion

### Sequencing results and quality control

Summary statistics related to reads can be seen in Table 1. Read quality was found to be excellent, with lower quartile Phred scores above 32 through to the 101st base (Additional file 1: Figure S1). The mean GC content of reads, 39 %, is consistent with values observed in previous EST-based investigations, for example [14], where 38.54 % content was found. In the first 5 positions in our reads a number of sequences were found to be over-represented, likely to be a result of bias in adaptor hexamer binding as previously noted in other Illumina-based sequencing experiments [28] as no residual adaptor sequence was observed in our tests of the raw data.

### Assembly and completeness of dataset

**Fig. 3:**
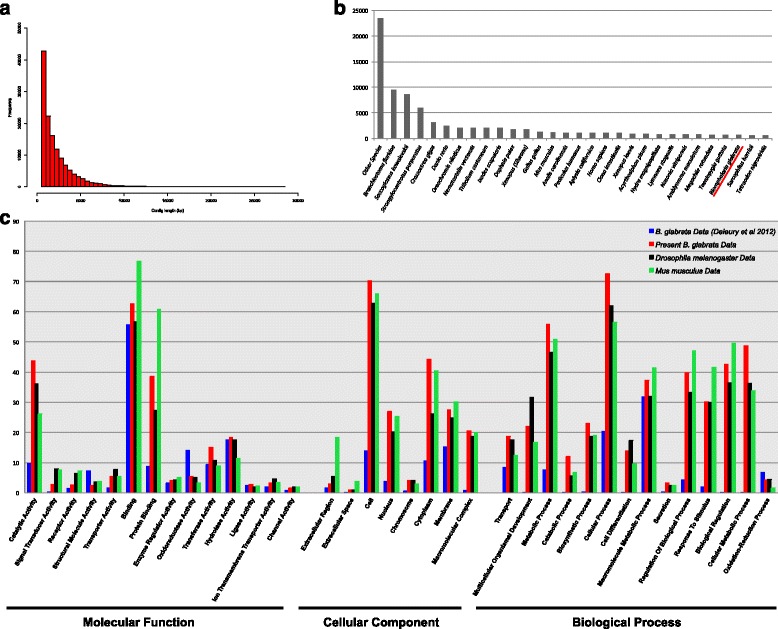
Analysis of the composition of the transcriptome of *B. glabrata*. **a** Contig length distribution graphed using R. BLAST2GO used to compare the distribution of BLASTx hits vs the nr database by best BLAST hit by species (**b**) and by GO category (**c**)

Statistics on the final assembly can be seen in Table 2, with a graph of the distribution of sequence lengths seen in Fig. 3a. The compressed fasta file with our transcriptome assembly (≥500 bp) can be downloaded from Figshare with the doi 10.6084/m9.figshare.3117385, and the untrimmed assembly (≥100 bp) is available from 10.6084/m9.figshare.3117406 (to download directly, type https://dx.doi.org/ before the doi reference). In our 500 bp minimum length assembly we recover 295 Mbp of transcribed sequence, which provides a deeper resource to draw from than heretofore available in this species. With 90,315 contigs greater than 1 kb in length and an N50 of 3,221 bp, the dataset is well-assembled and will reliably span complete domains, allowing for easy identification of genes.

**Table 2:** Basic assembly data: Summary of the metrics of assembly used in the present study

*Assembler:*	Trinity (*k* mer 25), 500bp min length
Number of contigs	133,084
Max contig length (bp)	28,281
Mean contig length (bp)	2219.48
Median contig length (bp)	1,537
N50 contig length (bp)	3,221
# contigs in N50	27,526
# contigs > 1kb	90,315
# bases, total	295,377,631
# bases in contigs > 1kb	265,182,009
GC Content %	38.04 %

Assessment with the Core Eukaryotic Genes Mapping Approach (CEGMA) set of 248 ultra-conserved core eukaryotic genes (CEGs) found in nearly all eukaryotic organisms [42] identified 247 of these as present in our dataset. This represents 99.6 % coverage, a figure comparable or better than most complete genomic sequencing projects, and suggests that this transcriptomic resource provides an almost total recovery of the basic genetic repertoire of this species.

### Comparison with extant resources

While sequencing efforts on the *B. glabrata* genome are still underway, transcriptomic resources are vital for gaining an understanding of the molecular processes behind aspects of this species’ disease resistance, development and physiology. To date, the most complete resource available, [14], is a fairly comprehensive transcriptomic resource, assembled from 30 adult snails, ranging from juvenile (4-7 mm shells) to older adult (12-16 mm shells) BgBRE individuals, from Illumina reads. However, this transcriptome does not sample as broadly across the course of development as the one presented here, and suffers according to many standard metrics of assessment. For example, the N50 of the Dheilly et al. resource, 1,067 bp according to our calculation, is threefold less than that of the assembly described here (3,221 bp). Given that 30,206 sequences were assigned a GO identity in the Dheilly et al. assembly, c.f. 74,492 in ours, the additional length of our assembly also is advantageous in allowing more firm homology assignment to be made.

To assess the degree of overlap between our sample and the Dheilly et al. resource we then used BLASTn (with the Megablast setting) to detect the number of sequences shared in common between the two assemblies. Of the 326,874 sequences in the ‘transcriptsBre1et2.fasta’ file, 241,019 (73.73 %) were found in our 500 bp minimum size assembly, and 268,903 (82.26 %) in our original, untrimmed dataset. Reciprocally, of the 133,084 contigs in our assembly, 119,264 (89.62 %) have at least a partial hit in the Dheilly et al. resource. We therefore recover the majority of the Dheilly et al. assembly in our data, with markedly more complete length as discussed earlier. We suspect that much of the portions of the transcriptomic resources that do not overlap are temporally or spatially restricted in expression, with those in our assembly representing embryonically transcribed genes, and those in Dheilly et al. more likely to be adult-specific.

### Functional annotation and analysis

BLAST2GO was used to functionally annotate this data for comparison with other species and previously completed *B. glabrata* GO distributions [13]. The results of this analysis can be seen in Fig. 3b and c, and full GO annotations are available in Additional file 2.

As a result of the excellent assembly statistics, a large number of identifiable sequences were obtained. Of the 133,084 contigs in our dataset, 80,952 (60.8 %) possess a homologue in the nr database (BLASTx, *E* value cut off 10^-3^). Comparison of the BLASTx best hits results by species reveals that, despite the accessioning of oyster *C. gigas* and limpet *L. gigantea* genomic data onto the nr database, the closest hits gained using BLASTx are more heavily weighted towards deuterostome species. These species have previously been noted as having a slow rate of molecular evolution, and it may be this resultant similarity in sequence, compared with the fast-evolving Ecdysozoan species similarly well represented in the nr database, which causes this result.

Of the sequences with a BLAST-annotatable homologue in the nr database, 74,492 were mapped and of these 53,412 were assigned GO terms. The distribution of several GO assignment distributions in the three second-level functional categories (biological process, cellular component and molecular function), can be seen in Fig. 3c. In concert with our CEGMA results, we are therefore confident that this dataset contains the sizeable majority of transcribed RNA in this species, although it is likely a number of RNAs of low copy number and restricted temporal expression are not present.

The GO terms shown in Fig. 3c correspond to those shown in [13], a previously published annotated *B. glabrata* transcriptomic dataset. This allows comparison of the results of BLAST2GO analysis between our data, previously extant data for this species, and the distributions of the proteomes of the well-described model organisms *D. melanogaster* and *M. musculus.* The distributions seen in Fig. 3c suggest that our dataset is more representative of the GO term distributions seen in complete proteomes than those available previously in *B. glabrata*. While some differences are to be expected in the distribution of GO identities from species to species and GO distribution is an imperfect measure of the completeness of a dataset, our results, in red, mirror those of the fully sequenced species much more closely than those of the previously available *B. glabrata* datasets shown in blue (Fig. 3c).

### Pathway mapping and completeness

Along with CEGMA and GO-based evidence, KEGG pathway mapping suggests we have near-100 % coverage of all major signaling and metabolic pathways, with well-conserved processes such as gluconeogenesis and the citrate cycle demonstrating complete coverage of all their constituent steps. The coverage of these, and other key pathways, can be seen in Table 3, and all annotations can be seen in Additional file 3. Generally, greater than 90 % coverage can be seen for all genes expected in protostome metazoans (NB, KEGG pathways shown also show genes restricted to other clades).

**Table 3:** Coverage of a range of key conserved metabolic pathways: Coverage of components of a selection of key conserved and/or disease associated pathways, as mapped by the KAAS-KEGG automatic annotation server

KO Pathway ID	KO pathway name	KEGG components expected in protostomes (total possible^a^)	KEGG KASS mapped *B. glabrata* homologues	% Expected pathway covered
00010	Glycolysis/Gluconeogenesis	31 (57)	28	90 %
00020	Citrate cycle TCA cycle	22 (27)	22	100 %
00071	Fatty acid metabolism	61 (84)	60	98 %
00280	Valine, Leucine and Isoleucine degradation	47 (56)	47	100 %
04210	Apoptosis	47 (60)	36	77 %
04310	Wnt signaling pathway	65 (71)	58	89 %
04330	Notch signaling pathway	23 (24)	20	87 %

^a^Expected = component # found in any other protostome species in the KEGG database (Total also includes those only found in other lineages, e.g. Prokaryota). Please note: repeats of same gene in KEGG map are included in total count

**Fig. 4:**
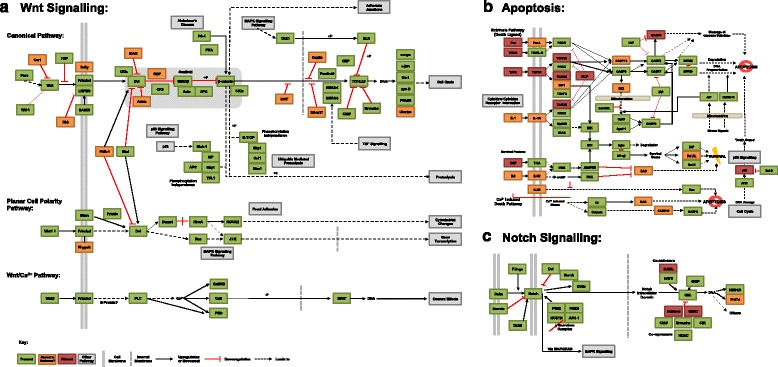
KEGG pathways for Wnt (**a**), apoptosis (**b**), and Notch (**c**) signalling pathways, showing presence of majority of components in our transcriptome, in green. Where components were not identified by the KEGG SBH annotation process, this is indicated in orange (where these are not known in the Spiralia to date) or red (when these would be expected). Absence may be real or the result of marked sequence divergence between genes in our dataset and those orthologs in the KEGG database

To illuminate the coverage provided by our dataset and its utility in reconstructing cellular processes involved in immune responses and developmental signalling, the Wnt, apoptosis and Notch signaling pathways are shown in Fig. 4. Apoptosis is a key part of molluscan disease response [50] and the Wnt and Notch pathways are involved in a wide range of patterning mechanisms in the process of growth and development.

While coverage of the KEGG apoptosis pathway map is the least well-recovered of the pathways shown here, at 77 % it is still remarkably complete, and contains many prospective targets for future research. The differences in orthology between spiralian genes and the more well-researched vertebrate paralogs (after the whole genome duplications observed in that lineage) will require careful unteasing. For example, protostomes exhibit far less CASP gene diversity, but sub- and neo-functionalisation in vertebrates may mean that single *B. glabrata* orthologues likely play the role of several paralogous sequences from the Vertebrata. The recovery of greater than three quarters of this pathway in *B. glabrata* here nevertheless represents a considerable advance in our knowledge of this pathway in the Mollusca in general, and in *B. glabrata* in particular.

Similarly, the Wnt and Notch signaling pathways are well-recovered in our dataset and are representative of similar signaling cascades not shown in figures here, but available in Additional file 3. Such recovery in key conserved signaling cascades suggests that our dataset could also be of utility for a range of further investigations in fields such as endocrinology, developmental and cell biology.

It should also be noted that some genes may be present in our dataset, but be considerably different in sequence to the query database used to identify genes, which currently contains a very limited number of spiralian gene sequences. This would lead to under-reporting of completeness, and lends further weight to hypothesized excellent coverage in our dataset in known gene pathways.

### Disease-related pathway components

To assay the potential utility of this transcriptomic dataset as a resource for investigation of disease processes, we have targeted particular gene families for manual annotation. We are able to recover a range of disease-response associated genes in our transcriptome, including a number of genes from families not before identified in *B. glabrata*. These genes are listed in Table 4, and their complete sequences can be found in Additional file 4.

**Table 4:** Manual annotation of disease-response associated genes

Gene	# Orthologues	# Isoforms/allelic variants
*BgRel*	1	10
*BgRelish*	1	11
*nuclear factor of activated T cells*	2	2 and 6
*CREB*	1	2
*STAT*	1	19
*FREPs, CREPs and GREPs*	see text	81

Molluscs generally utilize innate immune responses, such as cell-mediated reactions, to fight infection, as they appear to lack the adaptive immune system found in vertebrates. Numerous gene families were also examined and found to be present in this resource. *Nuclear factor kappa-light-chain-enhancer of activated B cells* (*NF- κβ*) genes are found in all metazoan lineages, and are noted as "rapid-acting" primary transcription factors, capable of quick response to harmful stimuli [19]. Two *NF- κB* have been previously published in *B. glabrata*: *BgRel* (ACZ25559.1) and *BgRelish* (ACZ25560.1) [59]. Both of these sequences are found in our dataset, with several forms of *BgRelish* gene identified (split between Comp98599, which possesses eight potential isoforms, and Comp97091, with three sequences recovered). Several variants of the *BgRel* gene were noted in our sample (Comp89844, four isoforms, and Comp91314, six isoforms). Two potential *nuclear factor of activated T cells* genes were also noted in our sample, Comp99436 (two isoforms) and Comp99884 (6 isoforms), which provide an additional target for functional work in this well-conserved gene family, and further suggests a role for Toll-like receptor (TLR) and immune deficiency (IMD)-like pathways in immune response.

CREB (cAMP response element-binding protein) and STAT (signal transducer and activator of transcription) genes play roles in proliferation and phagocytosis respectively [59]. Using known *B. glabrata* sequences as queries (tBLASTn, *E* < 10^-10^), homologues of these genes are found in at least two and 19 contigs respectively in our dataset, and these sequences can be seen in Additional file 4, which will allow further work complementing previous investigations in this species [59]. The functional role for these novel sequences are as yet uncharacterized in *B. glabrata* or the Mollusca in general, but with the description of this complement, further investigation is possible.

While the genes noted here represent only a small fraction of the likely *B. glabrata* immune response cassette and our report of these is intended to be indicative of the utility of our dataset rather than a conclusive treatment of the scope of these molecules, their number and ready identifiability nevertheless underlines the utility of the dataset presented here as a tool for the identification of the components of such pathways. There are numerous other immune-related pathways that could be investigated using the data presented, and this resource therefore stands as a good resource for ongoing work in this species, especially until the advent of a complete genomic resource.

### FREP, CREP and GREP immunoglobulin superfamily containing genes

Along with other components of innate immune responses, molluscs are known to utilise fibrinogen-encoding proteins (FREPs) as part of their immune defences [1, 58]. These contain immunoglobulin superfamily domains and can therefore be recognized even in *de novo* datasets [60]. Recent RNAseq-based efforts [14] have uncovered two related families of genes related to FREPS, known a C-type lectin-related proteins (CREPs) and Galectin-related protein (GREPs) which also are believed to play a part in mediating these responses. Collectively, these molecules are known as Variable Immunoglobulin and Lectin domain containing molecules (VIgL). The transcriptomic resource described here contains 81 contigs with high similarity (tBLASTn, *E* < 10^-9^) to known *B. glabrata* VIgL proteins, along with many of slightly less strong homology. While complete mapping of the VIgL families is beyond the scope of this manuscript, this resource adds significantly to the number of these genes which can be investigated in detail for their responses to schistosome infection in the future.

### Identifying differentially expressed genes in extant datasets

A large number of previous studies have compared infected *B. glabrata* with uninfected controls, using microarray and Expressed Sequence Tag (EST) based techniques, but have been hamstrung by an inability to discern the identity and function of gene sequences found to be differentially expressed due to the short length of EST sequences. These studies often contain information from a range of sample types, and a large amount of useful data, which may be of vital utility in the fight against schistosomiasis, can be gleaned from them with the aid of a more complete transcriptomic resource.

For example, [56] used a microarray-based method to identify 98 differentially expressed sequences from haemocytes of schistosome-resistant and schistosome-susceptible *B. glabrata* after exposure to excretory-secretory products from *S. mansoni.* Of these 98 EST derived sequences, 61 were at that time unable to be assigned homology to known gene families due to their short length, and therefore were excluded from further analysis in that manuscript. However, using BLASTn (cutoff *E* < 10^-6^ *,* although this value generally = 0.0) we were able to recognize 20 more of these EST sequences within our transcriptomic dataset. With their greater average length (mean = 1359.9 bp) we were able to determine that one contig in our dataset (comp100220_c2_seq2) corresponded to five of the ESTs included in the list of 61 unknown sequences. This contig can, with the aid of the complete sequences provided by our dataset, be recognized as belonging to a single, long *alpha-actinin A* like-sequence, whose prevalence in earlier EST-based studies might indicate a cytoskeletal response to schistosome infection.

Furthermore, the additional sequence provided by our contigs allowed more of these unknown sequences to be more firmly identified. Of the 20 newly identifiable EST sequences (including all five matching comp100220_c2_seq2), only four were unable to be matched to known sequences in Genbank using BLASTx. In almost all cases, the best hits were also to *B. glabrata* sequence provisionally accessioned by other studies, with homologous genes in other species matching less well. Of these 16 ESTs identifiable courtesy of our resource, 5 are similar to *alpha-actinin A* as noted above. Others include *gastric intrinsic factor, serine/arginine-rich splicing factor 7* and *NRDE-2.* These genes also may be useful targets in the treatment of schistosomiasis, and our dataset can in this way act as a bridge between previous functional investigations such as [56] and sequences of presently unknown function in the nr database.

## Conclusions

In this paper we have presented a deep transcriptomic resource for the medically important species *B. glabrata,* gleaned from samples taken at a variety of life stages. This resource has been shown to contain an almost entire cassette of genes from a variety of conserved pathways, suggesting it is a nearly complete sampling of the transcribed *B. glabrata* RNA complement. Crucially, given *B. glabrata*’s role as a vector for schistosomiasis, the deep transcriptomic resource presented here will allow a range of biomedical investigations to take place and further allow research into invertebrate immune systems, an area where our knowledge is still nascent at best. Evidence shown here suggests that this dataset will be a reservoir for drawing further understanding from previous work as well as acting as a baseline for ongoing research. The dataset presented here will therefore stand as a useful resource for the assessment of patterns of evolution within the Mollusca, for human health, and in many other spheres, both immediately and well into the future.

## Additional files

Additional file 1: Figure S1. FastQC output: a) Read quality and b) Nucleotide %age distribution by read position. (PDF 258 kb)
Additional file 2: Annotations of transcriptome. (GZ 1986 kb)
Additional file 3: KEGG annotations and pathway maps. (GZ 23266 kb)
Additional file 4: Sequences of all genes referred to in text. (XLSX 130 kb)

## Abbreviations

BLAST: Basic local alignment search tool
CEG: Core eukaryotic genes
GO: Gene ontology
NCBI: National Centre for Biotechnology Information
nr: non redundant
