# Supplementary figures and images for: Deep, multi-stage transcriptome of the schistosomiasis vector Biomphalaria glabrata provides platform for understanding molluscan disease-related pathways
Source: BMC Infect Dis. 2016 Oct 28;16:618. doi: 10.1186/s12879-016-1944-x (PMC5084317; doi:10.1186/s12879-016-1944-x)

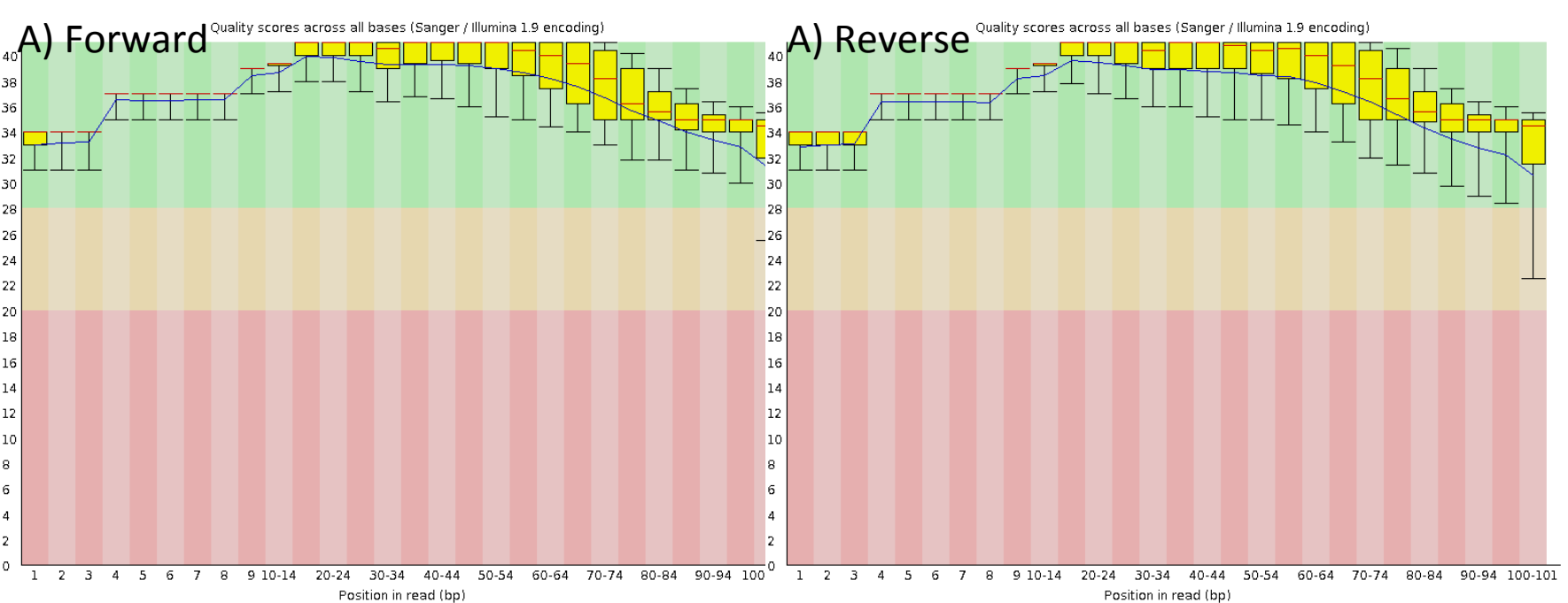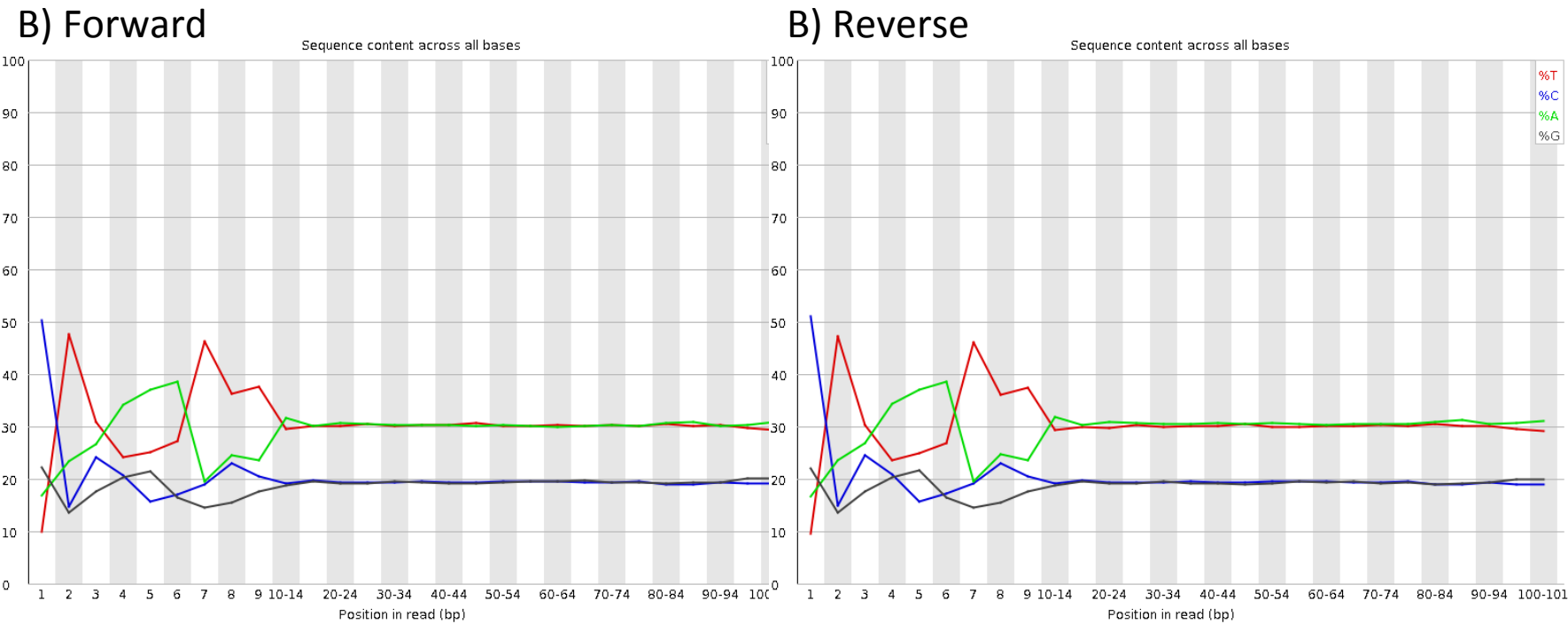

Supplement: Additional file 1: Figure S1. — FastQC output: a) Read quality and b) Nucleotide %age distribution by read position. (PDF 258 kb) [file 12879_2016_1944_MOESM1_ESM.pdf]
